# Cloning of the GABA_B_ Receptor Subunits B1 and B2 and their Expression in the Central Nervous System of the Adult Sea Lamprey

**DOI:** 10.3389/fnana.2016.00118

**Published:** 2016-12-08

**Authors:** Daniel Romaus-Sanjurjo, Blanca Fernández-López, Daniel Sobrido-Cameán, Antón Barreiro-Iglesias, María Celina Rodicio

**Affiliations:** Department of Functional Biology, CIBUS, Faculty of Biology, Universidade de Santiago de CompostelaSantiago de Compostela, Spain

**Keywords:** GABA, GABA_B1_, GABA_B2_, agnathans, receptor heterodimerization

## Abstract

In vertebrates, γ-aminobutyric acid (GABA) is the main inhibitory transmitter in the central nervous system (CNS) acting through ionotropic (GABA_A_) and metabotropic (GABA_B_) receptors. The GABA_B_ receptor produces a slow inhibition since it activates second messenger systems through the binding and activation of guanine nucleotide-binding proteins [G-protein-coupled receptors (GPCRs)]. Lampreys are a key reference to understand molecular evolution in vertebrates. The importance of the GABA_B_ receptor for the modulation of the circuits controlling locomotion and other behaviors has been shown in pharmacological/physiological studies in lampreys. However, there is no data about the sequence of the GABA_B_ subunits or their expression in the CNS of lampreys. Our aim was to identify the sea lamprey GABA_B1_ and GABA_B2_ transcripts and study their expression in the CNS of adults. We cloned two partial sequences corresponding to the GABA_B1_ and GABA_B2_ cDNAs of the sea lamprey as confirmed by sequence analysis and comparison with known GABA_B_ sequences of other vertebrates. In phylogenetic analyses, the sea lamprey GABA_B_ sequences clustered together with GABA_B_s sequences of vertebrates and emerged as an outgroup to all gnathostome sequences. We observed a broad and overlapping expression of both transcripts in the entire CNS. Expression was mainly observed in neuronal somas of the periventricular regions including the identified reticulospinal cells. No expression was observed in identifiable fibers. Comparison of our results with those reported in other vertebrates indicates that a broad and overlapping expression of the GABA_B_ subunits in the CNS is a conserved character shared by agnathans and gnathostomes.

## Introduction

γ-aminobutyric acid (GABA) is the main inhibitory transmitter in the central nervous system (CNS), and it acts via ionotropic (GABA_A_) and metabotropic (GABA_B_) GABA receptors (Pinard et al., 2010; Chalifoux and Carter, 2011). Unlike GABA_A_ receptors that form ion channels, GABA_B_ receptors address second messenger systems through the binding and activation of guanine nucleotide-binding proteins [G-protein-coupled receptors (GPCRs)], producing a slower and more prolonged inhibition than ionotropic GABA_A_ receptors, which results in reduced neuronal excitability (Blein et al., 2000; Bettler et al., 2004; Pinard et al., 2010). GABA_B_ receptors have been identified on both pre- and postsynaptic terminals (Curtis et al., 1968, 1977; Alford and Grillner, 1991). Presynaptic GABA_B_ receptors can exist as either auto-(those that control GABA release) or heteroreceptors (those activated by other neurons) to modulate neurotransmitter release (Bettler et al., 2004). Depending on whether GABA_B_ receptors are activated on excitatory or inhibitory terminals, their effects on the postsynaptic neuron are inhibitory or dis-inhibitory, respectively. Currently, it is accepted that functional GABA_B_ receptors are obligate heterodimers (Kammerer et al., 1999; Kuner et al., 1999; Blein et al., 2000; Villemure et al., 2005; Geng et al., 2013) composed of two receptor subunits, GABA_B1_ (Kaupmann et al., 1997; Padgett and Slesinger, 2010) and GABA_B2_ (Kaupmann et al., 1998; White et al., 1998; Kuner et al., 1999). The GABA_B1_ receptor subunit is responsible for agonist binding (Galvez et al., 2001), whereas the GABA_B2_ receptor subunit is essential for trafficking of the heterodimer to the cell surface and for signal transduction following agonist activation (Calver et al., 2002) through the activation of G-proteins (Villemure et al., 2005). Interestingly, a study of Maurel et al. (2008) indicates a possible formation of GABA_B_ oligomers, which show decreased G-protein coupling efficiency. Thus, formation of GABA_B_ oligomers may regulate receptor efficacy, making their formation potentially critical to cellular function.

The presence of GABA_B_ receptors in a single-cell species of paramecium has been demonstrated by immunohistochemical methods (Ramoino et al., 2006), which indicates that the GABA_B_ receptor appeared early during evolution. Only a few studies have shown the presence and/or expression of GABA_B_ receptors in invertebrate species. An optogenetic study revealed GABA_B_ receptor expression in motor neurons of *Caenorhabditis* *elegans* (Schultheis et al., 2011). Ramoino et al. (2007) demonstrated a GABAergic-like system as well as the expression of GABA_B1_ and GABA_B2_ subunits in a marine demosponge, *Chondrilla nucula*. Expression of GABA_B_ receptors has been reported in olfactory sensory neurons of moths (Pregitzer et al., 2013) and in the entire CNS of cockroaches (Blankenburg et al., 2015), spiders (Panek et al., 2003) and *Drosophila melanogaster* (Mezler et al., 2001). The expression of GABA_B_ transcripts (GABA_B1_ and GABA_B2_) has been also reported in a few jawed vertebrate species (rats: Bischoff et al., 1999; Fritschy et al., 1999; humans: Calver et al., 2000; Berthele et al., 2001; non-human primates: Muñoz et al., 1998, 2001; zebrafish: Tabor et al., 2008; and frogs: Kaeser et al., 2011). These studies reveal a wide distribution of this receptor in the entire CNS of invertebrate and vertebrate species. So far, the expression of the GABA_B_ subunits has not been reported in any jawless vertebrate. It has been suggested that more studies providing a detailed distribution of the GABA_B1_ and GABA_B2_ subunits are necessary to understand the roles that these receptors have in CNS neurotransmission (Charles et al., 2001).

The sea lamprey, *Petromyzon marinus* L., belongs to an ancient lineage of vertebrates, the Agnathans or jawless vertebrates, which occupy a key phylogenetic position at the transition between urochordates and jawed vertebrates. Lampreys are important model vertebrates for studies of nervous system development and evolution, as its genome is a key reference to understand molecular evolution in vertebrates (Smith et al., 2013). Sea lampreys have a complex and long life cycle, which includes a metamorphosis that transforms blind filter-feeder larvae into young adults (post-metamorphic sea lampreys). These descend to the sea to feed as parasites of teleost fishes and then, after 1–2 years, mature adult sea lampreys return to rivers to breed and die (Hardisty and Potter, 1971).

In addition, lampreys have been extensively used to identify the cellular mechanisms involved in the generation and control of vertebrate locomotion (Dubuc et al., 2008; Grillner et al., 2008). Many pharmacological and physiological studies have shown the importance of GABA in the modulation of central pattern generators (CPGs) and sensory inputs in the spinal cord (Tegnér et al., 1993; Parker et al., 1998; Schmitt et al., 2004), and brain circuits that control locomotion, including the paleostriatal pathway and the striatal neurons projecting to the motor centers (Grillner et al., 2008); as well as the specific role of GABA_B_ receptors in these processes (Alford and Grillner, 1991). Lampreys have become an interesting model to understand successful regeneration following spinal cord injury (reviewed in Barreiro-Iglesias, 2012, 2015; Rodicio and Barreiro-Iglesias, 2012). A recent study of our group demonstrated that in lampreys, as in mammals, there is a massive release of glutamate and GABA after a complete spinal injury (Fernández-López et al., 2014). GABA accumulated in the form of *halos* around some of the descending axons after the injury and statistical analyses showed a correlation between the presence of this *halos* and a higher survival ability of the identified descending neurons (Fernández-López et al., 2014). This revealed a possible neuroprotective role of GABA following spinal cord injury in lampreys. This type of studies show the importance of increasing our knowledge on the GABAergic system of lampreys.

In lampreys, the distribution of GABA immunoreactive neurons and fibers in the CNS of adults (Meléndez-Ferro et al., 2000; Robertson et al., 2007), their ontogeny (Meléndez-Ferro et al., 2002, 2003; Ruiz et al., 2004), the origin of the descending GABAergic projections to the spinal cord (Valle-Maroto et al., 2011) and the co-localization of GABA with others classic neurotransmitters in the same neuron (Villar-Cerviño et al., 2008, 2010, 2013, 2014; Barreiro-Iglesias et al., 2009a,b) have been widely studied. However, and despite the wide knowledge on the GABAergic system of lampreys, the cloning and molecular characterization of the GABA_B_ receptors subunits of lampreys as well as their pattern of expression in the CNS have not been reported so far.

In the present study, we report the identification and characterization, by means of phylogenetic and sequence analyses, of sea lamprey sequences corresponding to GABA_B1_ and GABA_B2_ cDNAs. We also report the pattern of expression of these transcripts in the brain and spinal cord of young (post-metamorphic) and mature (upstream migrating) adults of the sea lamprey. Moreover, we compare the expression pattern of the two GABA_B_ transcripts in the sea lamprey with those of other species, providing an anatomical and genetic basis to further understand the roles that this pre- and post-synaptic receptor may have in mediating inhibitory neurotransmission in the CNS.

## Materials and Methods

### Animals

Young (*n* = 4) and mature adults (*n* = 4) of the sea lamprey, *Petromyzon marinus* L., were used for the *in situ* hybridization experiments. Ten larvae were used for RNA extraction. Young adults and larvae were collected from the River Ulla (Galicia, Northwest Spain) and mature upstream migrating adults were obtained from a local commercial supplier. All experiments were approved by the Bioethics Committee of the University of Santiago de Compostela and the Consellería do Medio Rural e do Mar of the Xunta de Galicia (JLPV/IId) and conformed to the European Union and Spanish regulations for the care and handling of animals in research.

### Cloning and Sequencing of Sea Lamprey GABA_B1_ and GABA_B2_ Partial cDNAs

Larvae were anesthetized by immersion in 0.1% ethyl 3-aminobenzoate methanesulfonate salt (MS-222; Sigma, St. Louis, MO, USA) and the brain and spinal cord were dissected out under sterile conditions. Total RNA was isolated from these tissues using the TriPure reagent (Roche, Mannhein, Germany). The first-strand cDNA synthesis reaction from total RNA was catalyzed with Superscript III reverse transcriptase (Invitrogen, Waltham, MA, USA) using random primers (hexamers; Invitrogen). For polymerase chain reaction (PCR) cloning, specific oligonucleotide primers, 5′-TGGCACTGGCCCTGAACAAG-3′ forward and 5′- GTTGAGGTTGGGCTGCGAGT-3′ reverse; and 5′- GACAAATCTTGCTCGACGCC-3′ forward and 5′- AAACGTTGCTGAGGACACCA-3′ reverse, were designed based on the sea lamprey GABA_B1_ and GABA_B2_ sequences, respectively, annotated in the sea lamprey genome and deposited in the Ensembl database (Smith et al., 2013,^1^). The amplified fragments were cloned into pGEM-T vectors (Promega, Madison, WI, USA) and sequenced by GATC Biotech (Cologne, Germany) using Sanger sequencing. Sequence analysis and comparison was done through Basic Local Alignment Search Tool (BLAST) on NCBI and in the SMART website (Schultz et al., 1998; Letunic et al., 2015,^2^).

### Phylogenetic Analysis

For this analysis, GABA_B1_ (protein GenBank ID: *Canis lupus familiaris*: XP_005640226.1; *Drosophila melanogaster*: AAK13420.1; *Danio rerio*: CAP09588.1; *Bos taurus*: AAI46242.1; *Homo sapiens*: AAH50532.2; *Mus musculus*: AAH54735.1; *Rattus norvegicus*: CAE84069.1;* Columba livia*: XP_013227121.1; *Xenopus laevis*: ADQ43745.1;* Pan troglodytes*: XP_009449053.1) and GABA_B2_ (protein GenBank ID: *Canis lupus familiaris*: XP_538749.2; *Danio rerio*: NP_001137515.1; *Drosophila melanogaster*: AAK13421.1; *Columba livia*: XP_013227167.1; *Homo sapiens*: AAH35071.2; *Mus musculus*: NP_001074610.1; *Rattus norvegicus*: EDL98850.1; *Xenopus laevis*: ADQ43746.1; *Pan troglodytes*: XP_009455264.1; *Bos taurus*: XP_002689780.1) sequences of representative species were aligned with the sea lamprey GABA_B1_ and GABA_B2_ partial deduced protein sequences deposited in the Ensembl database. All sequences were aligned using CLUSTALW and a phylogenetic tree was constructed using the neighbor-joining method with Poisson corrected distances on amino acids and bootstrap analysis (1000 replications) using MEGA 6 software (Tamura et al., 2013). An alternative phylogenetic tree was constructed with the minimum-evolution method with Poisson corrected distances on amino acids and bootstrap analysis (1000 replications) using also the MEGA 6 software.

### *In situ* Hybridization

Templates for *in vitro* transcription were prepared by PCR amplification as follows. Two 459 base pairs (bp) and 446 bp fragments corresponding to GABA_B1_ and GABA_B2_ sequences, respectively, were obtained using the primers mentioned above. In this case, the reverse primers include the sequence of the universal T7 promoter (TAAGCTTTAATACGACTCACTATAGGGAGA). For the generation of sense probes, the sequence of the T7 promoter was included in the forward primers. The identity of the amplified fragments was confirmed by direct sequencing. Digoxigenin (DIG)-labeled riboprobes were synthesized using the amplified fragments as templates and following standard protocols using T7 polymerase (Roche Diagnostics, Germany).

*In situ* hybridization experiments were performed as previously described for riboprobes against the serotonin 1a receptor (5-ht1a; Cornide-Petronio et al., 2013). Briefly, the brain/rostral spinal cord of young and mature adults were fixed by immersion for 12 h in 4% paraformaldehyde. Then, they were cryoprotected with sucrose 30% and sectioned in a cryostat in the transverse plane. Two parallel series of sections were obtained. The sections of each series were incubated with the GABA_B1_ or GABA_B2_ DIG-labeled probes, respectively, at 70°C overnight in hybridization mix and treated with RNAse A (Invitrogen) in the posthybridization washes. Then, the sections were incubated with a sheep anti-DIG antibody conjugated to alkaline phosphatase (1:2000; Roche) overnight. Staining was conducted in BM Purple (Roche) at 37°C until the signal was clearly visible. Finally, the sections were mounted in Mowiol^®^ (Calbiochem; Temecula, CA, USA) and photographed with an Olympus photomicroscope (AX-70; Provis) equipped with a color digital camera (Olympus DP70; Tokyo, Japan). Images were slightly adjusted for brightness and contrast with Adobe Photoshop CS4 to compose the plates. No staining was observed when sense probes were used.

## Results

### Characterization and Phylogenetic Analysis of the Sea Lamprey GABA_B_ Subunits

**Figure 1:**
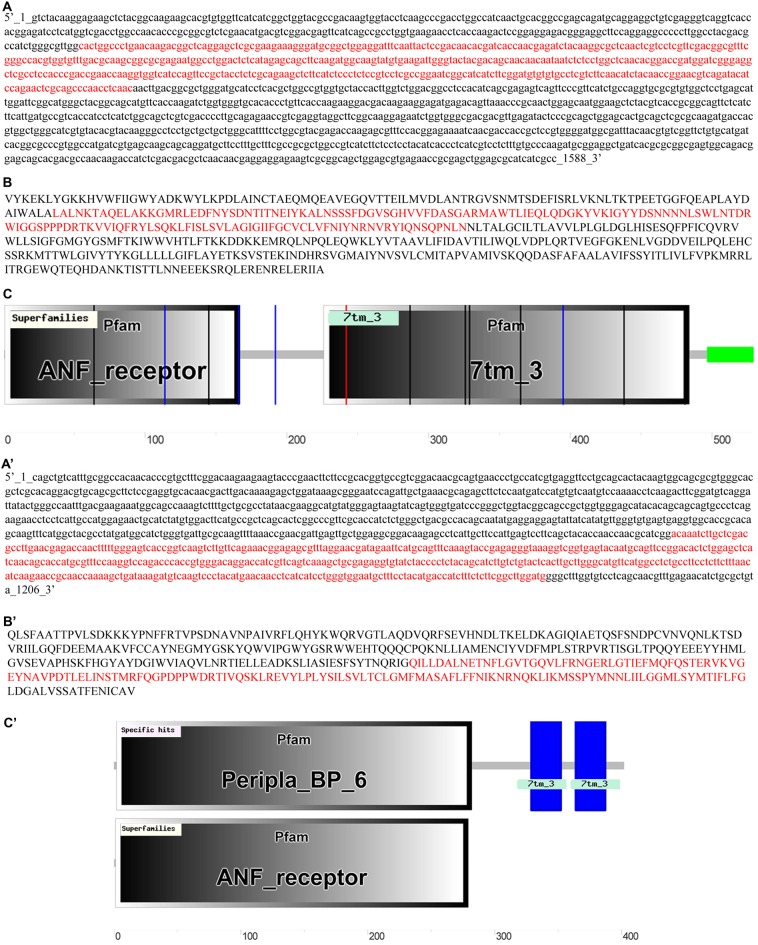
**Partial cDNA sequences of the GABA_B1_** **(A)** and GABA_B2_ **(A′)** subunits from the Ensembl database, with the cloned sequences marked in red. Sequences of the deduced GABA_B1_ **(B)** and GABA_B2_ **(B′)** proteins with the sequence corresponding to the cloned cDNA sequences highlighted in red. Analyses of the predicted GABA_B1_ **(C)** and GABA_B2_ **(C′)** proteins in SMART and Basic Local Alignment Search Tool (BLAST).

The cloned GABA_B1_ and GABA_B2_ cDNAs sequences corresponded to the nucleotides 302–760 for GABA_B1_ (Figure 1A), and 729–1162 for GABA_B2_ (Figure 1A′) of the partial cDNA sequences of these receptors annotated in sea lamprey genome of the Ensembl database. The cloned sequences had a 100% similarity with the putative GABA_B1_ and GABA_B2_ sequences of the Ensemble database, respectively. The cloned sequences were deposited in the GenBank database (GABA_B1_: KX655780; GABA_B2_: KX655781). Due to a high level of similarity between the cloned sequences and those annotated in the Ensembl database, we used the partial, but longer, GABA_B1_ and GABA_B2_ protein sequences (Figures 1B,B′, respectively) deduced from the sea lamprey genome sequences ENSPMAG00000006844 (corresponds to GABA_B1_), which is located in the scaffold GL479777, and ENSPMAG00000004383 (corresponds to GABA_B2_), which is located in the scaffold GL478877, for the subsequent analyses. Both partial deduced protein sequences contained the “ligand-binding domain of GABA_B_ receptors” and the “seven transmembrane sweet-taste receptor of 3 GCPR” as revealed by the SMART online tool (Figures 1C,C′).

Percentages of similarity between the sea lamprey GABA_B1_ and GABA_B2_ partial deduced protein sequences and those of other species are shown in Table 1. We observed a percentage of similarity of 68% to 73% and 66% to 69% between the sea lamprey partial GABA_B1_ and GABA_B2_ deduced protein sequences, respectively, and the corresponding deduced protein sequences of other vertebrate species. As expected, the percentage of similarity of the partial sea lamprey GABA_B1_ and GABA_B2_ deduced amino acids sequences with those of the protostome invertebrate *D. melanogaster* is low (less than 50%). The similarity between the sea lamprey partial GABA_B1_ and GABA_B2_ deduced protein sequences is only 33%.

**Table 1:** **Percentage of homology between the partial amino acid sequences of the sea lamprey GABA_B_ subunits and the amino acid sequences of GABA_B_ subunits of different vertebrate and invertebrate species**.

GABA_B1_	*Petromyzon marinus* (%)
*Xenopus laevis* (ADQ43745.1)	73
*Homo sapiens* (AAH50532.2)	72
*Mus musculus* (AAH54735.1)	72
*Pan troglodytes* (XP_009449053.1)	72
*Bos taurus* (AAI46242.1)	71
*Canis lupus* (XP_005640226.1)	71
*Columba livia* (XP_013227121.1)	71
*Danio rerio* (CAP09588.1)	71
*Rattus norvegicus* (CAE84069.1)	68
*Drosophila melanogaster* (AAK13420.1)	48

GABA_B2_	*Petromyzon marinus* (%)

*Canis lupus* (XP_538749.2)	69
*Columba livia* (XP_013227167.1)	69
*Homo sapiens* (AAH35071.2)	69
*Pan troglodytes* (XP_009455264.1)	69
*Mus musculus* (NP_001074610.1)	68
*Rattus norvegicus* (EDL98850.1)	68
*Bos taurus* (XP_002689780.1)	67
*Xenopus laevis* (ADQ43746.1)	67
*Danio rerio* (NP_001137515.1)	66
*Drosophila melanogaster* (AAK13421.1)	41

**Figure 2:**
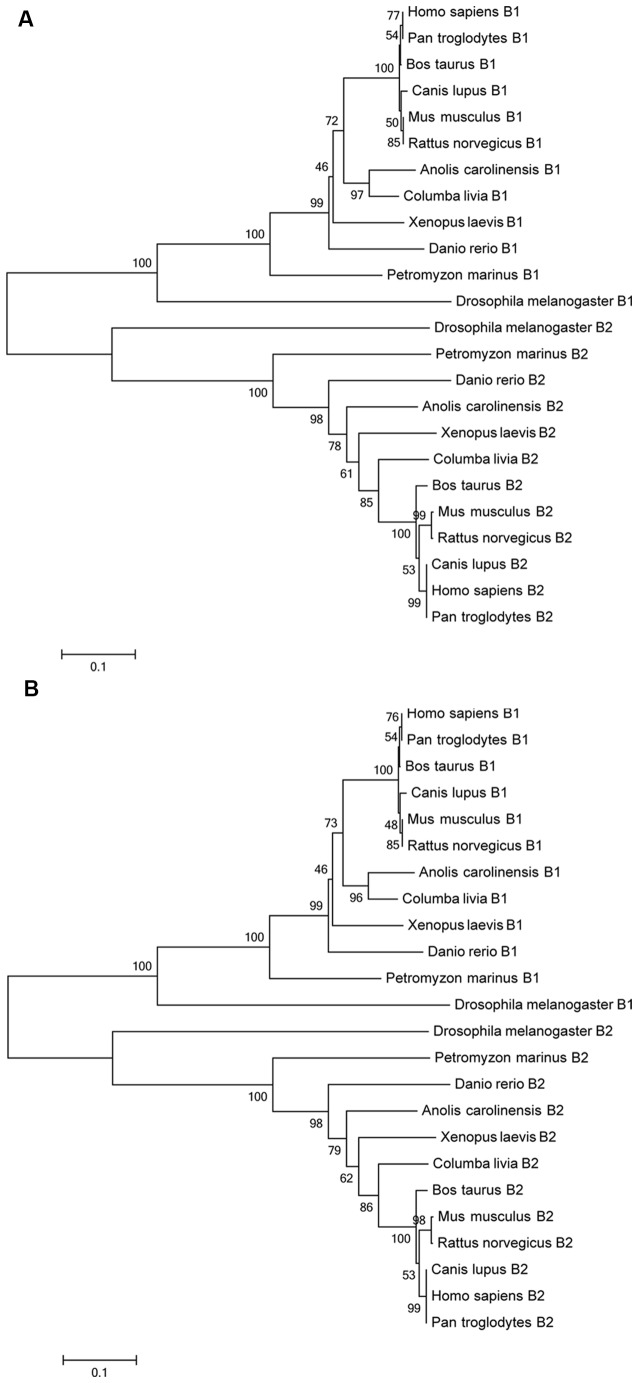
**Phylogenetic trees of GABA_B_ protein sequences of different species according the neighbor-joining (A)** and the minimum-evolution methods **(B)**. The bootstraps are indicated on a scale of 100 based on 1000 replications. Scale indicates 0.1 amino acids substitutions per locus.

The GABA_B1_ and GABA_B2_ deduced protein sequences annotated in the Ensembl database were aligned with GABA_B1_ and GABA_B2_ deduced protein sequences of representative vertebrate and invertebrate species and this alignment was used to construct a phylogenetic tree using the neighbor-joining and the minimum-evolution methods. All vertebrate GABA_B1_ and GABA_B2_ sequences, including the sea lamprey sequences, were clustered together in their respective groups in the resulting trees and the sea lamprey sequences were found to emerge as an outgroup to the corresponding gnathostome sequences (Figures 2A,B).

### Expression of GABA_**B1**_ and GABA_B2_ Subunits in the Adult Sea Lamprey Brain

We observed a widespread expression of the GABA_B1_ and GABA_B2_ transcripts in neuronal populations in the brain of the adult sea lamprey. No clear and obvious differences were observed in the expression of these transcripts between young and mature adult sea lampreys. *In situ* hybridization signal appeared as a dotted labeling in the tissue sections.

**Figure 3:**
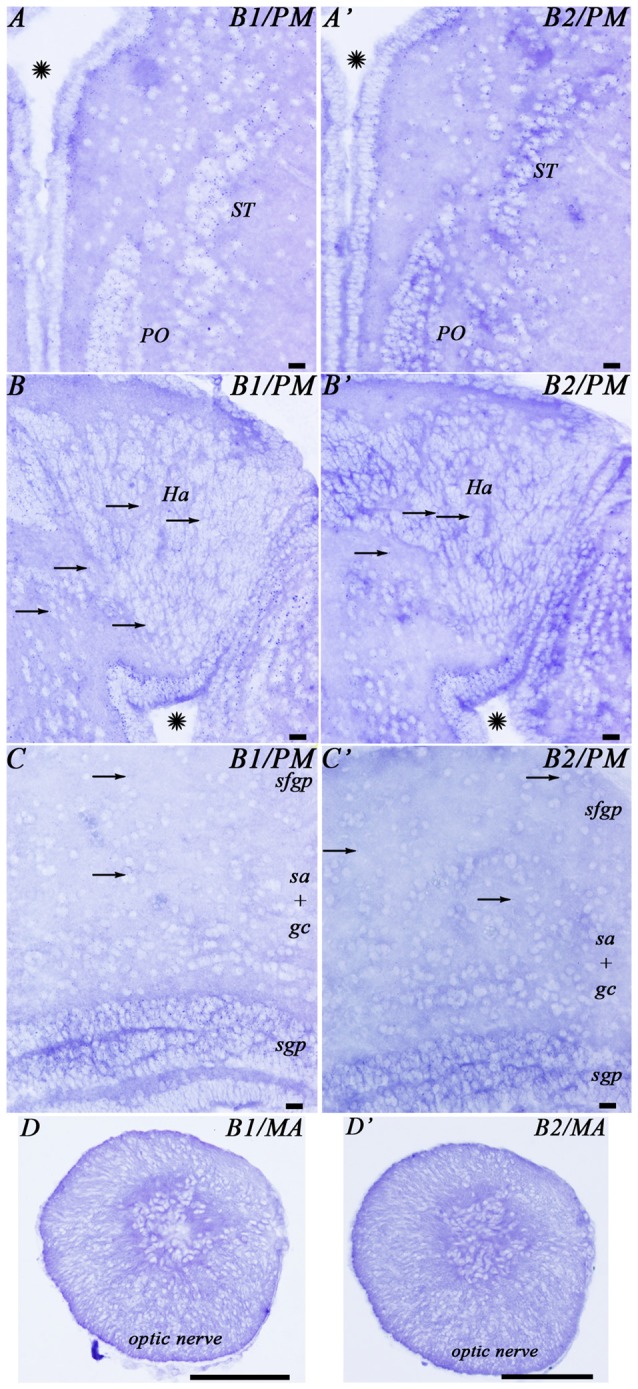
**Photomicrographs from the sea lamprey brain showing the expression of both GABA_B_ subunits in the same brain regions. (A–D)** corresponds to GABA_B1_ expression and **(A′–D′)** corresponds to GABA_B2_ expression. The arrows indicate some small positive dots. Please note in **(D,D′)** that the astrocytes are located in the central part of the optic nerve. Asterisks indicate the ventricles. Abbreviations as in Figure 4. Scale bars = 50 μm.

**Figure 4:**
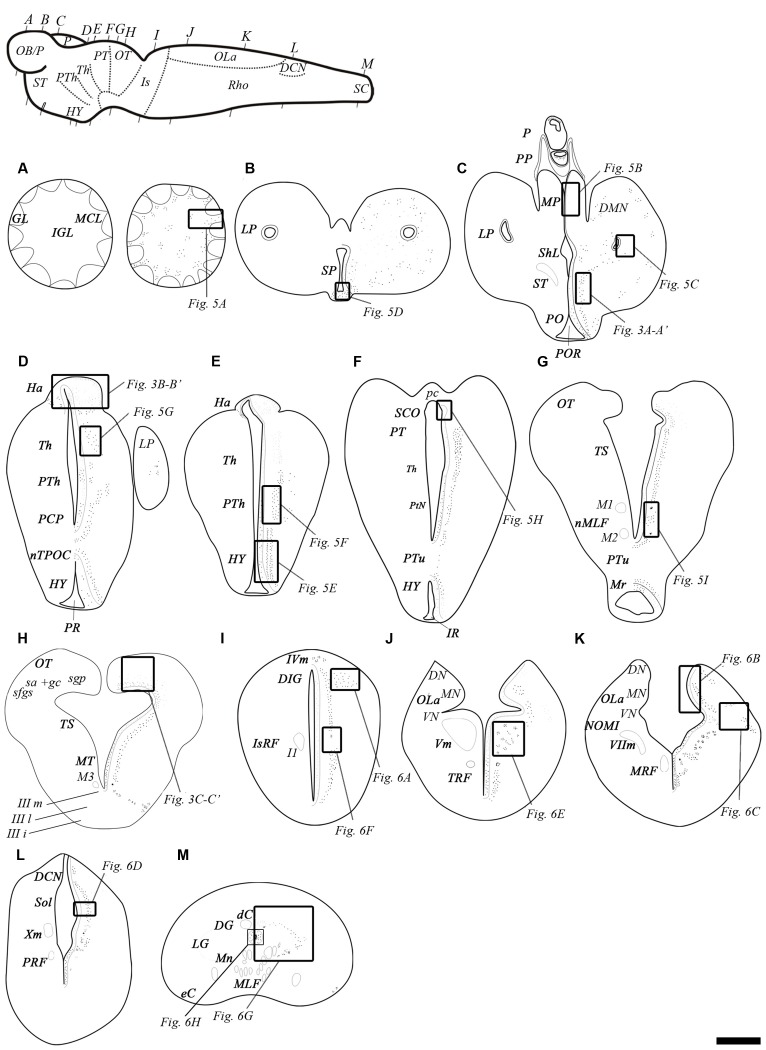
**Schematic drawings of transverse sections of the adult sea lamprey brain and spinal cord showing the distribution of GABA_B_ transcripts (on the right side), and the main brain and spinal cord regions (on the left side).** **(A–G)** Representative sections of the prosencephalon in a rostro-caudal order. **(G,H)** Representative sections of the mesencephalon in a rostro-caudal order. Note that section **(G)** includes rostral mesencephalic and caudal diencephalic regions. **(I–L)** Representative sections of the rhombencephalon in a rostro-caudal order. **(M)** Representative section of the spinal cord. The level of sections is indicated in the upper left figure showing a lateral view of the brain. Dots represent the location and an estimate of the relative density of both GABA_B_ transcripts. Correspondence with photomicrographs in other figures is indicated by squared areas. Abbreviations: dC, dorsal cell; DCN, dorsal column nucleus; DG, dorsal gray; DIG, dorsal isthmic gray; DMN, dorsomedial neuropil; DN, dorsal nucleus of the octavolateral area; eC, edge cell; GL, glomerular layer; Ha, habenula; HY, hypothalamus; I1, isthmic Müller cell 1; IGL, inner granular layer; IIIi, intermediate oculomotor subnucleus; IIIl, lateral oculomotor subnucleus; IIIm, medial oculomotor subnucleus; IR, infundibular recess; IsRF, isthmic reticular formation; IVm, trochlear motor nucleus; LP, lateral pallium; LG, lateral gray; M1, Müller cell 1; M2, Müller cell 2; M3, Müller cell 3; MA, mature upstream migrating adult; MCL, mitral cell layer; MLF, medial longitudinal fasciculus; MN, medial nucleus of the octavolateral area; Mn, motor neurons; MP, medial pallium; Mr, mammilar region; MRF, middle rhombencephalic reticular formation; MT, mesencephalic tegmentum; nMLF, nucleus of the medial longitudinal fasciculus; NOMI, intermediate octavomotor nucleus; nTPOC, nucleus of the tract of the postoptic commissure; OB/P, olfactory bulbs/pallium; OLa, octavolateral area; OT, optic tectum; P, pineal organ; pc, posterior commissure; PCP, paracommissural preoptic nucleus; PM, post-metamorphic sea lamprey; PO, preoptic nucleus; POR, preoptic recess; PP, parapineal organ; PR, postoptic recess; PRF, posterior rhombencephalic reticular formation; PT, pretectum; PTh, prethalamus; PtN, posterior tubercular nucleus; PTu, posterior tuberculum; sa + gc, stratum “album” et griseum central; SCO, subcommissural organ; sfgs, stratum fibrosum et griseum superficiale; sgp, stratum griseum periventriculare; ShL, subhippocampal lobe; Sol, nucleus of the solitary tract; SP, septum; ST, striatum; Th, thalamus; TRF, trigeminal reticular formation; TS, torus semicircularis; Vm, trigeminal motor nucleus; VIIm, facial motor nucleus; VN, ventral nucleus of the octavolateral area; Xm, vagal motor nucleus. Scale bars = 50 μm.

**Figure 5:**
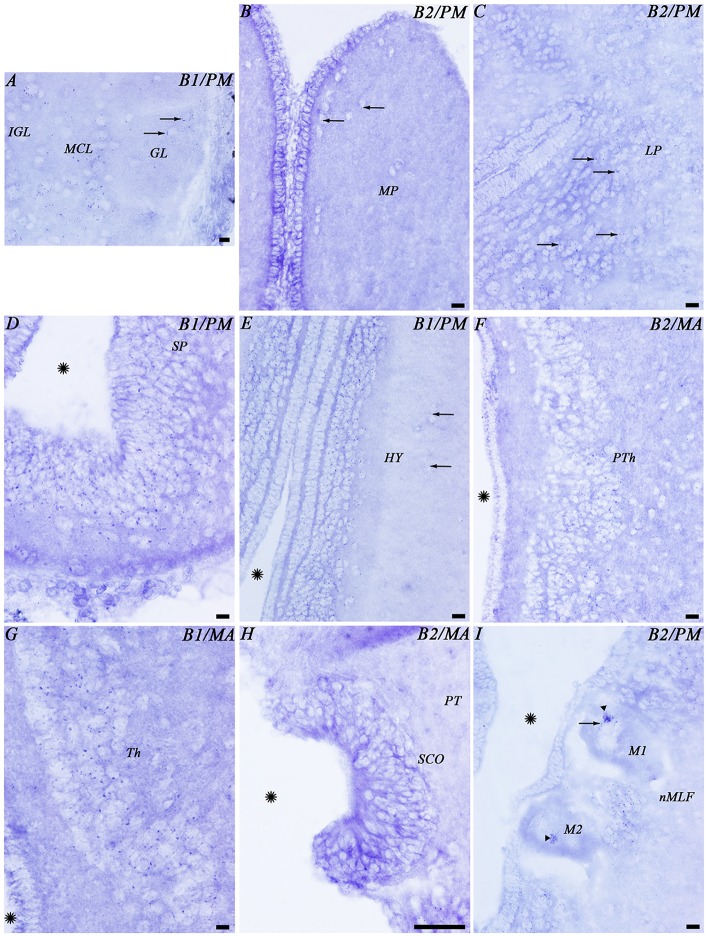
**Photomicrographs from different regions of the prosencephalon showing expression of both GABA_B_ transcripts.** Abbreviations as in Figure 4. **(A–I)** Photomicrographs of the forebrain regions indicated in Figure 4. **(A,D,E,G)** GABA_B1_. **(B,C,F,H,I)** GABA_B2_. Arrows indicate small positive dots. Arrowheads indicate accumulations of transcript expression in giant cells. Asterisks indicate the ventricle. Scale bars = 50 μm.

**Figure 6:**
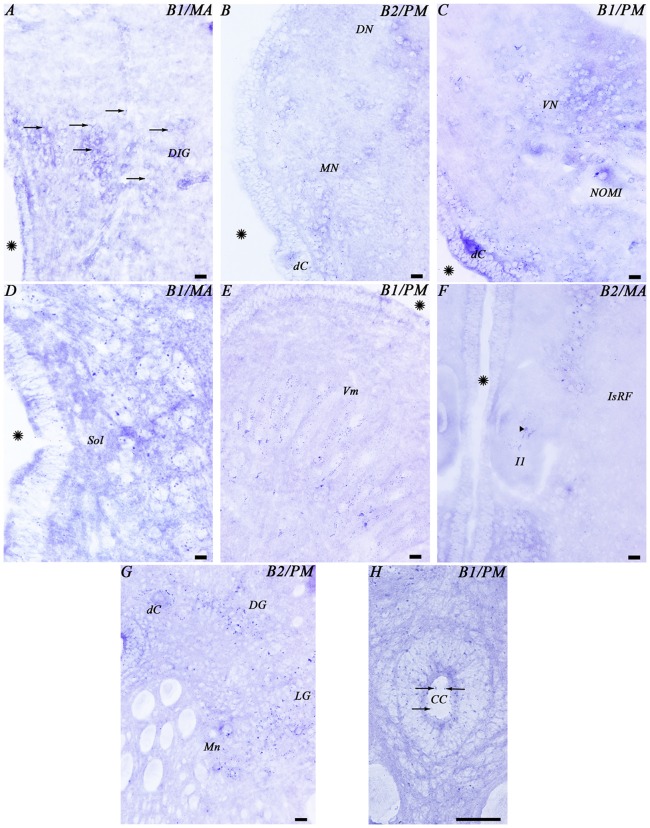
**Photomicrographs from different regions of the mesencephalon** **(A,B)**, rhombencephalon **(C–F)** and spinal cord **(G,H)** showing expression of both GABA_B_ transcripts. Abbreviations as in Figure 4. Arrows indicate small positive dots. The arrowhead indicates accumulations of transcript expression in a giant cell. Asterisks indicate the ventricle. Scale bars = 50 μm.

No clear differences were noted in the expression of the transcripts of the two GABA_B_ subunits (Figure 3). Accordingly, only 1 set of schematic drawings of transverse sections taken from a young post-metamorphic adult showing the expression pattern of both transcripts is presented in Figure 4. Photomicrographs of representative transverse sections of the brain are shown in Figures 3, 5, 6. These photomicrographs were taken from two young postmetamorphic and three upstreaming migrating adults.

In the telencephalon, the olfactory bulbs showed numerous dots of positive GABA_B1_ and GABA_B2_ expression (Figures 4A, 5A). Most of the expression was observed in cells of the mitral and inner cellular layers. Some expression was also observed in cells localized between the glomeruli. In the medial pallium, GABA_B1_ and GABA_B2_ expression was very scarce in cells of the periventricular layer and no expression was observed in migrated cells (Figures 4C, 5B). We observed expression of both transcripts in neurons surrounding the dorsomedial neuropil (Figure 4C). The lateral pallium showed more GABA_B1_ and GABA_B2_ expression in the cells of the periventricular layer than in migrated cells (Figures 4B,C, 5C). In the subhippocampal lobe, most of the GABA_B1_ and GABA_B2_ expression was observed in the cells of the periventricular layer (Figure 4C). In the subpallium, the cells of the septum showed GABA_B1_ and GABA_B2_ positive dots (Figures 4B, 5D). In the striatum, GABA_B1_ and GABA_B2_ expression was observed mainly in migrated cells, with less expression being observed in the periventricular layer (Figures 3A,A′, 4C). In contrast, the preoptic nucleus showed wide GABA_B1_ and GABA_B2_ expression in periventricular cells, mainly in the intermediate layer of the preoptic nucleus (Figures 3A,A′, 4C).

The hypothalamus showed wide expression of the GABA_B1_ and GABA_B2_ transcripts in cells of the periventricular layers, while the migrated cells showed very scarce expression (Figure 5E). Many GABA_B1_ and GABA_B2_ positive dots were observed in the nucleus of the postoptic commissure, the paracommissural preoptic nucleus, the dorsal and ventral hypothalamus and the mammillary nucleus (Figures 4D–F, 5E). The pattern of expression observed in the hypothalamus was also observed in the prethalamus and thalamus (Figures 4D–F, 5F,G). GABA_B1_ and GABA_B2_ expression was also observed in the pineal and parapineal organs (Figure 4C). The habenula showed GABA_B1_ and GABA_B2_ expression, with some differences between the left and right sides (Figures 3B,B′, 4D,E). In the right habenula, which is bigger than the left one, GABA_B1_ and GABA_B2_ positive dots were more numerous than in the left habenula, although positive dots in cells of the left habenula were larger. The pretectum showed expression of both transcripts in cells of the periventricular layer and scarce expression in migrated neurons (Figure 4F). In the paratubercular nucleus, very few cells showed expression of the transcripts (Figure 4F). In contrast, a large number of GABA_B1_ and GABA_B2_ positive dots were observed in the nucleus of the medial longitudinal fascicle including the first and second Müller cells (Figures 4G, 5I).

In the mesencephalon, the optic tectum showed GABA_B1_ and GABA_B2_ positive dots with most of them distributed throughout the cells of the stratum griseum periventriculare, but also in cells of the stratum “album” et griseum central (Figures 3C,C′, 4H). The torus semicircularis showed numerous positive dots. The positive dots in cells of the torus were larger than those in the optic tectum (Figure 4H). The migrated neurons of both areas showed very little expression of the transcripts. There was strong GABA_B1_ and GABA_B2_ expression in the tegmentum and the mesencephalic reticular area (including the third Müller cell) and the motor neurons of the oculomotor nuclei showed a large amount of GABA_B1_ and GABA_B2_ expression in their somas (Figure 4H).

In the alar plate of the rhombencephalon, the dorsal isthmic gray showed expression of both transcripts (Figures 4I, 6A). In the octavolateral nuclei, there was GABA_B1_ and GABA_B2_ expression, mainly in cells of the periventricular layer (Figures 4J,K, 6B,C). The octavomotor nuclei showed GABA_B1_ and GABA_B2_ positive dots (Figures 4K, 6C). Numerous GABA_B1_ and GABA_B2_ positive dots were observed in cells of the periventricular layer of the solitary tract (Figures 4L, 6D) and dorsal column nuclei. Dorsal cells of the caudal rhombencephalon (primary medullary and spinal nucleus of the trigeminus) also showed GABA_B1_ and GABA_B2_ expression (Figures 6B,C). In the basal plate of the rhombencephalon, the soma of the cells of the visceromotor nuclei (V, VII, IX and X) showed many GABA_B1_ and GABA_B2_ positive dots (Figures 4J–L, 6E). In addition, wide GABA_B1_ and GABA_B2_ expression was observed in all rhombencephalic reticular nuclei (isthmic, trigeminal, middle and posterior reticular nuclei), including the giant Müller and Mauthner cells (Figures 4I–L, 6F).

### Expression of the GABA_B1_ and GABA_B2_ Subunits in the Spinal Cord

In the rostral spinal cord of the adult sea lamprey, most neuronal types showed GABA_B1_ and GABA_B2_ expression (Figures 4M, 6G,H). Dorsal cells showed large amounts of GABA_B1_ and GABA_B2_ positive dots in their soma (Figures 4M, 6G). We also observed some expression of the transcripts in the edge cells (Figure 4M). GABA_B1_ and GABA_B2_ expression was observed in interneurons of different sizes located in the dorsal and lateral regions (Figures 4M, 6G). Expression of both transcripts was observed in the somas of motor neurons (Figure 6G). Cerebrospinal fluid-contacting (CSF-c) cells showed numerous GABA_B1_ and GABA_B2_ positive dots, even in their apical dendrites (Figure 6H).

### Expression of GABA_B1_ and GABA_B2_ Subunits in Glial Cells

The glial cells of the brain of the sea lamprey are almost exclusively ependymal cells, although astrocytes could be identified in the optic nerve. No expression of any of the two transcripts was observed in brain ependymocytes (examples in Figures 5B,C) or in the subcommissural organ (Figures 4F, 5H). The astrocytes of the optic nerve, which are easily identified because of the absence of neurons in the optic nerve, did not show expression of the transcripts (Figures 3D,D′).

## Discussion

### Relationships and Phylogeny of the Sea Lamprey GABA_**B**_ Genes and GABA_B_ Protein Sequences

Here, we report for the first time the identification and characterization of the GABA_B1_ and GABA_B2_ cDNA sequences and the distribution of the GABA_B_ transcripts in the CNS of the adult sea lamprey.

In our BLAST searches of the sea lamprey genome database, we found 1 GABA_B1_ gene and 1 GABA_B2_ gene, whose sequences were confirmed after PCR amplification and cloning of the respective cDNAs. The presence of only a single gene of each GABA_B_ subunit is also the case in mammals, birds and amphibians. Two paralogous copies of the GABA_B1_ subunit gene have been found in zebrafish (Klee et al., 2012), which is probably due to the additional whole-genome duplication that occurred in the actinopterygian lineage (Taylor et al., 2001). In the phylogenetic trees, the GABA_B1_ and GABA_B2_ partial amino acid sequences of the sea lamprey were located at the base of the vertebrate branches clustering the GABA_B1_ and GABA_B2_ sequences, appearing as sister members, respectively, of the GABA_B1_ and GABA_B2_ sequences of gnathostomes. Each of the subunits is grouped in an independent cluster (Figure 2). The location of the sea lamprey GABA_B1_ and GABA_B2_ sequences at the base of the vertebrate branches of the phylogenetic tree and its higher similarity with the vertebrate sequences than with those of *Drosophila* are in agreement with the phylogenetic position of lampreys and confirmed the GABA_B_ identity of the sea lamprey sequences identified in our study.

### Heterodimerization of the GABA_B_ Receptor

It is currently accepted that a functional GABA_B_ receptor consists of a heterodimer of GABA_B1_ and GABA_B2_ subunits (Jones et al., 1998; Kaupmann et al., 1998; White et al., 1998; Bettler et al., 2004). GABA_B1_ binds to GABA, while GABA_B2_ is needed to transmit the signal, because G-protein coupling is mediated via GABA_B2_ (Margeta-Mitrovic et al., 2000; Calver et al., 2001; Galvez et al., 2001; Pagano et al., 2001; Geng et al., 2013). Because of this, it seems clear that the co-expression of GABA_B1_ and GABA_B2_ mRNAs is necessary to form a functional GABA_B_ receptor. In our study, we observed an overlapping expression of the GABA_B1_ and GABA_B2_ mRNAs in all brain regions and in the spinal cord of the adult sea lamprey when using consecutive brain sections. Co-localization of both subunits in the same single cells of lampreys is clear in the giant individually identifiable reticulospinal neurons like the Mauthner and Müller neurons. This is also the case in *D. melanogaster*, where no differences were observed between the expressions of GABA_B_ subunits in *in situ* hybridization assays (Mezler et al., 2001). In zebrafish, a recent study using qPCR methods has shown that the two b1 and the b2 subunits are all expressed in the same brain regions, with the b1b and b2 being more represented than the b1a in some regions and in the brain as a whole (Cocco et al., 2016). Kuner et al. (1999) observed by analyzing serial rat brain sections, that GABA_B1_ and GABA_B2_ transcripts are widely expressed and that they also show considerable overlap in most regions of the brain, although in some regions the expression of the GABA_B1_ transcript was enriched. This suggests that a wide and highly overlapping expression of the GABA_B1_ and GABA_B2_ subunits is an ancestral and conserved character of vertebrates and invertebrates.

In the mouse brain, an association between the GABA_B2_ subunit and M2 muscarinic receptors has been shown which appears to enhance muscarinic signaling (Boyer et al., 2009). In lampreys, pharmacological treatments combined with electrophysiological studies have shown a role for muscarinic receptors in the modulation of the trigeminal-reticular pathway (Le Ray et al., 2004) and in the activation of reticulospinal neurons (Smetana et al., 2007). Moreover, the presence of cells immunoreactive for muscarinic receptors has been shown in the region of the posterior rhombencephalic reticular nucleus of lampreys (Smetana et al., 2007), a region that shows the expression of the GABA_B2_ transcript (present results). Whether the modulation of muscarinic receptors by the GABA_B2_ subunit also occurs in lampreys needs further investigation. This would show us whether this is an ancestral characteristic of vertebrates.

### Analysis of the Functional Significance of the GABA_B1_ and GABA_B2_ Expression Observed in the Central Nervous System of the Sea Lamprey

All brain regions and the spinal cord showed a broad expression of both GABA_B_ transcripts in the adult sea lamprey, which is in concordance with previous reports in invertebrates (e.g., *D. melanogaster* (Mezler et al., 2001), cockroaches (Blankenburg et al., 2015) or spiders (Panek et al., 2003)) and in jawed vertebrates (e.g., humans (Calver et al., 2000; Berthele et al., 2001), non-human primates (Muñoz et al., 1998; Nürnberger and Schöniger, 2001), rats (Bowery et al., 1987; Bischoff et al., 1999; Clark et al., 2000), birds (Veenman et al., 1994), frogs (Kaeser et al., 2011) and zebrafish (Tabor et al., 2008; Cocco et al., 2016)). Positive *in situ* signal in sea lamprey brain sections had a granular appearance probably due to low expression of these mRNAs in each single cell of the sea lamprey. Previous studies looking at the expression of different neurotransmitter receptors in lampreys have shown that the *in situ* hybridization signals appeared as a dotted labeling in sections of the CNS: serotonin receptor 1A (Cornide-Petronio et al., 2013, 2014), dopamine receptor D2 (Robertson et al., 2012; Fernández-López et al., 2015), and dopamine receptor D4 (Pérez-Fernández et al., 2016); suggesting that low expression levels are a common feature of different metabotropic neurotransmitter receptors.

The broad expression of these transcripts in the CNS suggests that this receptor is extensively used in the modulation of brain circuits in lampreys. The expression of both GABA_B_ transcripts in non-GABAergic cells, which can be identified by their size and/or location, such as the mitral cells of the olfactory bulbs, the giant reticulospinal neurons, the spinal motoneurons, the motoneurons of the visceromotor rhombencephalic nuclei, the primary sensory cells of the rhombencephalon and spinal cord or the edge cells of the spinal cord indicate that the GABA_B_ receptor plays a role in the modulation of the activity of non-GABAergic cells in the sea lamprey brain. The expression of GABA_B_ transcripts observed here is in agreement with the GABAergic modulation of spinal motoneurons and interneurons mediated by the GABA_B_ receptor as reported in previous pharmacological and electrophysiological studies (Alford and Grillner, 1991; Alford et al., 1991; Matsushima et al., 1993; Schmitt et al., 2004). Edge cells in the spinal cord are richly innervated by GABAergic fibers (Fernández-López et al., 2012) and recent work has shown that they are modulated by GABA (Svensson et al., 2013). Our results suggest that GABA could act trough the GABA_B_ receptor in the edge cells. The present results also support the idea of the role of the GABA_B_ receptor in the modulation of the lamprey respiratory network (Bongianni et al., 2006; Cinelli et al., 2014). Also, in agreement with our expression results, a modulatory action of GABA onto the pathway from the lateral columns to reticulospinal neurons has been suggested to be mediated by the GABA_B_ receptor (Vinay et al., 1998). These reticulospinal inputs are also regulated by peptidergic transmitters (Parker, 2000). Our study extends the number of neuronal populations known to be modulated through GABA_B_ signaling and opens the opportunity to conduct functional studies on the role of GABA and the GABA_B_ receptor in other circuits.

The broad expression of the GABA_B_ transcripts together with the broad distribution of GABAergic cells in the brain and spinal cord (Meléndez-Ferro et al., 2001; Robertson et al., 2007) suggests that the sea lamprey GABA_B_ receptor could modulate the activity of GABAergic cells. The expression of the GABA_B_ transcripts in periglomerular cells of the olfactory bulbs, which are mainly GABAergic (Meléndez-Ferro et al., 2001), or in the CSF-c cells of the spinal cord, which are mainly GABAergic as well (Rodicio et al., 2008; Villar-Cerviño et al., 2008; Fernández-López et al., 2012; Jalalvand et al., 2014), could potentially support this hypothesis. Results from pharmacological and electrophysiological studies have shown that inhibitory premotor interneurons respond to the application of GABA_B_ agonists (Alford and Grillner, 1991; Alford et al., 1991; Matsushima et al., 1993). Unfortunately, the different requirements of fixative for *in situ* hybridization and GABA immunohistochemistry precluded us from providing a definitive demonstration of the presence of GABA_B_ receptors in GABAergic cells of the sea lamprey. The present results show expression of the GABA_B_ transcripts in the dendrites of CSF-c cells of the spinal cord. Currently, some functions have been proposed for CSF-c neurons in controlling the composition of the CSF and releasing substances into the ventricular system (Vígh et al., 2004; Jalalvand et al., 2016). The GABA_B_ receptor in these cells could play a role for detecting GABA in the CSF. The detection of serotonin from the CSF has been also proposed for the 5-HT1A receptor due its expression in CSF-c dendrites (Cornide-Petronio et al., 2013). As stated above, the GABA_B_ receptor could modulate CSF-c cells acting as an autoreceptor and/or as a heteroreceptor trough synapses from other GABAergic cells.

### GABA_B1_ and GABA_B2_ Expression in Glial Cells

Our results show a lack of expression of the GABA_B_ transcripts in ependymocytes along the lamprey brain. The ependymal cells are the main glial type present in the brain of the sea lamprey, while astrocytes are only associated to some nervous tracts. However, the spinal cord shows both types of glial cells (Retzius, 1893). In contrast to jawed vertebrates, oligodendrocytes are not present in lampreys. The glial cells of lampreys do not display immunoreactivity to glial fibrillary acid protein (GFAP), but they express cytokeratins (Merrick et al., 1995). Few works have been done to study the expression of GABA_B1_ and GABA_B2_ mRNAs and/or GABA_B1_ and GABA_B2_ subunits in the ependymal layer of vertebrates. A pharmacological study of Corns et al. (2013) reported that only the GABA_A_, and not GABA_B_, receptor mediates the GABAergic responses in mammalian ependymal cells surrounding the central canal. More studies in other groups of vertebrates are necessary to determine the evolution of this character due to the large evolution distance between mammals and lampreys. In addition, the cells of the subcommissural organ, a special type of ependymal cells, also lack the GABA_B_ receptor both in lampreys, and in other vertebrates like teleosts, frogs and mammals (Jiménez et al., 2000; Saha et al., 2000; Nürnberger and Schöniger, 2001). Studies in these vertebrates have shown that the GABAergic responses in the subcommissural organ are also mediated by GABA_A_ receptors.

No expression of the GABA_B_ transcripts was observed in astrocytes of the optic nerve although previous studies have reported the presence of GABA_B_ subunits in astrocytes, of rodents (Charles et al., 2003; Oka et al., 2006; Luyt et al., 2007; Beenhakker and Huguenard, 2010), and the expression of GABA_B1_ and GABA_B2_ mRNAs in human astrocytes (Lee et al., 2011). Again, more studies are necessary in other groups of vertebrates to determine whether the lack of GABA_B_ expression in astrocytes is the ancestral condition or a derived character.

## Conclusion

In this study we show for the first time the distribution of the GABA_B_ transcripts in the CNS of the sea lamprey. The location of the lamprey GABA_B1_ and GABA_B2_ sequences in the phylogenetic trees and their average of similarity with those of other species are in agreement with the phylogenetic position of lamprey and confirms their identity. A wide and overlapping expression of both GABA_B_ transcripts was observed in neurons of the brain and spinal cord of the sea lamprey. In contrast, no expression of the transcripts was observed in the ependymal layer. The broad expression of the GABA_B_ transcripts in the neuronal populations of the CNS and their absence in ependymocytes is in agreement with that observed in other vertebrates.

## Author Contributions

DR-S, BF-L, AB-I and MCR contributed to the acquisition of experimental data, data analysis/interpretation and drafting of the manuscript. DS-C contributed to the acquisition of experimental data and data analysis/interpretation; AB-I and MCR contributed to the concept/design of the study. All authors have approved the final manuscript.

## Funding

This work was supported by grants from the Spanish Ministry of Science and Innovation-FEDER (BFU2010-17174), Spanish Ministry of Economy and Competitiveness-FEDER (BFU2014-56300-P) and Xunta de Galicia (GPC2014/030). ABI was supported by a grant from the Xunta de Galicia (2016-PG008).

## Conflict of Interest Statement

The authors declare that the research was conducted in the absence of any commercial or financial relationships that could be construed as a potential conflict of interest.

## Footnotes

^1^http://www.ensembl.org

^2^http://smart.embl-heidelberg.de/
